# Comparison of Suture Anchor Constructs in Arthroscopic Rotator Cuff Reconstruction: Assessing Clinical Outcome and Treatment Cost Variations

**DOI:** 10.3390/jcm14238412

**Published:** 2025-11-27

**Authors:** David Endell, Tim Schneller, Moritz Kraus, Markus Scheibel

**Affiliations:** 1 Department for Shoulder- and Elbow-Surgery, Schulthess Clinic, 8008 Zurich, Switzerland; david.endell@kws.ch (D.E.); moritz.kraus@kws.ch (M.K.); 2 Department for Teaching, Research and Development, Schulthess Clinic, 8008 Zurich, Switzerland; tim.schneller@kws.ch; 3 Centrum für Muskuloskeletale Chirurgie (CMSC), Charité-Universitätsmedizin Berlin, 10117 Berlin, Germany

**Keywords:** arthroscopic rotator cuff repair, cost-effectiveness, suture anchors

## Abstract

**Background**: Cuff reconstructions vary due to different technical approaches by suture anchor manufacturers, as well as different suture construct configurations. **Objectives**: The main aim of this study is to primarily compare clinical outcomes and secondarily observe cost-effectiveness by assessing suture construct configurations in arthroscopic rotator cuff repair (ARCR). **Methods**: Using a retrospective local registry, we included patients undergoing arthroscopic rotator cuff repair who had been implanted with different anchor configurations and different anchor manufacturers. Data analysis was conducted via multiple linear regression, primarily evaluating the relationship between clinical scores (OSS; SSV) and suture construct configurations, to analyze, monitor, and compare the postoperative clinical development. Total surgical costs were also obtained from the clinical billing department for analysis of various factors, including Adjusted Life Years (ALYs) and Incremental Cost-Effectiveness Ratio (ICER), while controlling for sex, tear severity, and age. **Results**: A total of 317 patients were included in the final analysis, with a mean age at surgery of 60.1 ± 10.8 years, with 58% of patients being male. According to the Gerber tear severity classification, 23% of patients had a partial tear, 59% had at least one full-thickness tear, and the remaining 18% had a massive tear. Using linear regression models, the analysis of changes in Quality-Adjusted Life Years (QALYs) as the dependent variable did not yield statistically significant results. The postoperative development of the measured clinical scores (SSV; OSS) did not show a significant difference comparing the two manufacturers (*p* = 0.11, *p* = 0.85). However, the model evaluating costs identified significant effects related to the type of anchor configuration and manufacturer. Regarding anchor configurations, utilizing anchor configuration 1 or 2 resulted in lower costs by up to CHF 254.51 compared to the reference anchor configuration 4 (*p* < 0.05), after controlling for age, sex, tear severity, and anchor configuration. **Conclusions**: The primary findings of this study indicate that although clinical outcomes are generally consistent across various rotator cuff reconstruction scenarios, while secondarily the cost implications can differ significantly and are mainly attributed to the differing numbers of anchors required for each configuration and price setting of the manufacturer. The study underscores the importance of suture construct configuration and manufacturer selection in controlling healthcare costs while maintaining quality patient care.

## 1. Introduction

Arthroscopic rotator cuff reconstruction (ARCR) is the most frequently performed orthopedic shoulder surgery, experiencing a significant rise in procedure rates in recent decades [1,2]. The economic burden of rotator cuff disease extends beyond direct medical costs to include lost productivity, disability payments, and reduced quality of life [3]. The ARCR procedure is widely recognized for its effectiveness in treating rotator cuff tears and is becoming increasingly popular due to advancements in surgical techniques and better patient outcomes [2,3,4,5]. Double-row (DR) rotator cuff repair techniques were developed to improve anatomical footprint restoration compared to single-row (SR) repairs, with adaptations evolving since the early 2000s. A major advancement was the transosseous-equivalent (TOE) technique, which uses a bridging suture configuration to enhance tendon-to-bone contact and biomechanical stability [6]. Previous research has focused on cost effectiveness regarding knotted versus knotless suture techniques [7].

Recent studies have again demonstrated that rotator cuff repair is generally cost effective compared to conservative management, with Incremental Cost-Effectiveness Ratios (ICERs) well below accepted thresholds [3]. However, the cost-effectiveness of specific anchor systems and configurations for the TOE technique in the European market remains understudied [8].

Given the substantial economic implications of anchor selection, there is a lack of comprehensive clinical and cost-utility analyses for inpatient care in Europe comparing different manufacturers and configurations in rotator cuff surgery. This study aims to provide evidence-based guidance for surgical decision-making by primarily comparing clinical results of different suture anchor configurations and differently manufactured anchor types. Secondarily, a cost-utility analysis also assesses cost differences in the previously described configurations.

## 2. Methods

Between June 2018 and October 2022, this retrospective cohort study included adult patients diagnosed with either partial- or full-thickness rotator cuff tear who underwent primary arthroscopic rotator cuff repair (ARCR) at Schulthess Clinic Zurich, Switzerland by the same two senior surgeons and either received a suture anchor construct (using various transosseous-equivalent suture anchor configurations) by Manufacturer A (Arthrex, Naples, FL, USA) or Manufacturer B (Stryker, Kalamazoo, MI, USA). Patients were routinely documented as part of a local clinic registry and subsequently enrolled in this cohort study after providing informed consent. The study protocol was approved by the institutional ethics committee.

Inclusion criteria included the following:

- Patients > 18 years old who underwent arthroscopic rotator cuff reconstruction;
- Patients diagnosed with partial or complete rotator cuff tears are indicated for arthroscopic repair;
- Patients consenting for surgical repair and consenting that his/her clinical data may be reused for research purposes;
- Patients who received suture anchors from either Manufacturer A or B, all performed by the same two surgeons.

Exclusion criteria included the following:

- Shoulder instability;
- Rotator cuff revision surgery or prior rotator cuff surgeries;
- Inability to provide consent;
- Substance use disorders and poor general health conditions;
- Any condition that does not allow a clinical check-up or completion of the questionnaires (e.g., due to neurological, mental, or metabolic diseases).

## 3. Intervention

**Figure 1:**
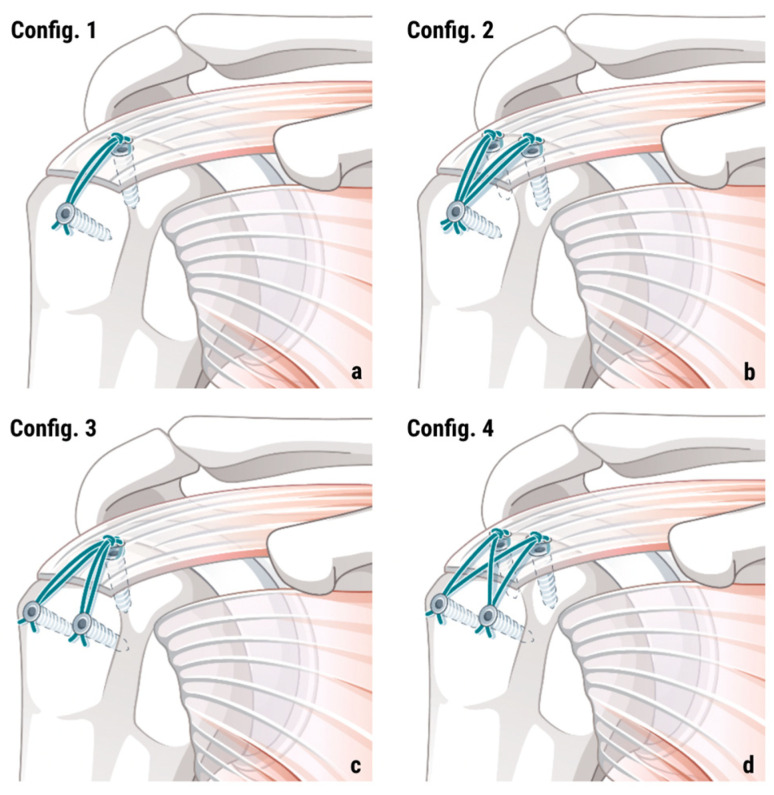
Overview of included TOE anchor configurations with 1 medial and 1 lateral anchor (config. 1 (**a**)), 2 medial and 1 lateral anchor (config. 2 (**b**)), 1 medial and 2 lateral anchors (config. 3 (**c**)), and 2 medial and 2 lateral anchors (config. 4 (**d**)).

Shoulder arthroscopies were conducted in accordance with standardized protocols and aligned with international guidelines, within the context of routine care and elective surgical procedures. Patients were positioned in the beach-chair position, and anesthesia was administered generally and additionally locally, as clinically indicated. Following diagnostic arthroscopy, the type and extent of rotator cuff pathology were assessed, including characterization of partial- or full-thickness tears, identification of involved tendons, and signs of degenerative changes. Concomitant intra-articular pathology was also evaluated. Ruptured rotator cuff tendons were mobilized to permit anatomical reduction to their native footprint, and tendon fixation was performed using various transosseous-equivalent suture anchor constructs (compare Figure 1), selected at the discretion of the operating senior surgeon. The anchors from Manufacturer A (Arthrex, Belp, Switzerland) consisted of the combination of 1–2 Corkscrew^®^ FT Anchors medially and 1–2 SwiveLock^®^-Anchors laterally. The anchor configuration of Manufacturer B (Stryker, Biberist, Switzerland) consisted of 1–2 Peek Zip Anchors medially and 1–2 ReelX STT Anchor laterally and represents a newer anchor generation with an incremental tension system for enhanced fixation of the lateral row. Anterolateral or lateral acromioplasty was performed when deemed necessary by the surgeon. Postoperative rehabilitation protocols followed the standardized protocol of the hospital and comprised an initial period of immobilization for 6 weeks, followed by progressive active-assisted and subsequently active, unresisted mobilization, beginning strengthening exercises 3 months after surgery.

## 4. Clinical Assessments

Patients underwent clinical evaluations before surgery (preoperative) and at the 6- and 24-month postoperative marks. All self-report data were assessed using REDCap, (Version 15.8.0) which is a free, secure, web-based application designed to support data capture for research studies [9].

The Oxford Shoulder Score (OSS) is a validated 12-item patient-reported questionnaire evaluating shoulder pain and function, with scores ranging from 12 (best) to 60 (worst) to evaluate shoulder pain and function [10].

The EQ-5D-5L is a standardized measure of health-related quality of life consisting of five dimensions (mobility, self-care, usual activities, pain/discomfort, anxiety/depression) with a utility index ranging from −0.66 to 1.0.

The Subjective Shoulder Value (SSV) is a self-report administered, reliable, responsive, and valid measure of shoulder function in patients undergoing SA that is highly correlated with other PROMs [11].

## 5. Data Analysis

A cost-effectiveness analysis was conducted to evaluate the economic impact of various anchor configurations and manufacturers. Multiple linear regression models were utilized to examine the relationship between total costs and factors such as Adjusted Life Years (ALYs), anchor type (manufacturer), anchor configuration, sex, tear severity, and age.

Throughout the study period (2018–2023), two types of surgical anchors were employed, which were introduced at different times and potentially influenced cost metrics due to differing market prices and operational characteristics. From 2018 through to part of 2021, Manufacturer A anchors were exclusively used. Starting in 2021 and continuing through to 2022, the hospital transitioned to using Manufacturer B anchors.

Incremental Cost-Effectiveness Ratios (ICERs) were estimated using a regression-based approach. The ICER represents the additional cost per additional unit of health benefit (quality-adjusted life year) and was calculated as follows:

ICER = (Cost_1_ − Cost_2_)/(Effect_1_ − Effect_2_)

where costs represent total direct medical costs and effects represent QALYs gained. A categorical variable representing anchor type was included in all statistical models to isolate the financial impact attributable to the manufacturer, independent of inflation-adjusted cost considerations.

Statistical analysis was performed using R software (Version 2023.06.2), with anchor manufacturer and configuration treated as categorical factors. Significance was set at *p* < 0.05, and the model’s fit was assessed via R-squared and F-statistics. Since the nature of our analysis was retrospective, including all available cases that met our inclusion criteria, a priori sample size calculation was not performed.

The study was performed in accordance with the standards of the Ethics Committee of Zurich (Kantonale Ethikkommission [KEK], Stampfenbachstrasse 121, CH-8090 Zurich, Switzerland), protocol code KEK-ZH-Nr. 2014–0253, date of approval: 18 August 2014, with amended protocol code PB_2020_00070, date of approval: 18 August 2020-rev, and with the 1964 Helsinki declaration and its later amendments or comparable ethical standards. All patients provided written informed consent prior to patient enrolment/data collection and use of their data for research purposes.

## 6. Results

The final analysis included 317 patients, with an overall mean age at surgery of 60.1 ± 10.8 years, and 58% of the cohort were male. According to the Gerber tear severity classification, 23% of patients had a partial tear, 37% had a single full tear, 22% had two or three torn tendons (only one full), and the remaining 18% had a massive tear. Patient demographics (see Table 1) were similar between manufacturer groups, with no significant differences in sex distribution or tear severity patterns (*p* > 0.05 for all comparisons). Age showed a significant difference (*p* = 0.03), but was adjusted for in the regression model.

The analysis of changes in Quality-Adjusted Life Years (QALYs) as the dependent variable did not yield statistically significant results (−0.94, *p* = 0.44).

**Figure 2:**
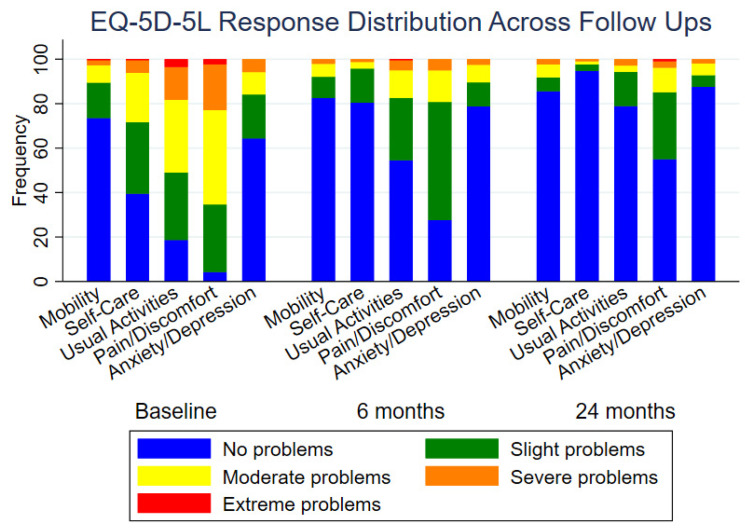
Changes in quality of life over time, showing measurements at baseline, 6 months, and 24 months post-intervention.

**Figure 3:**
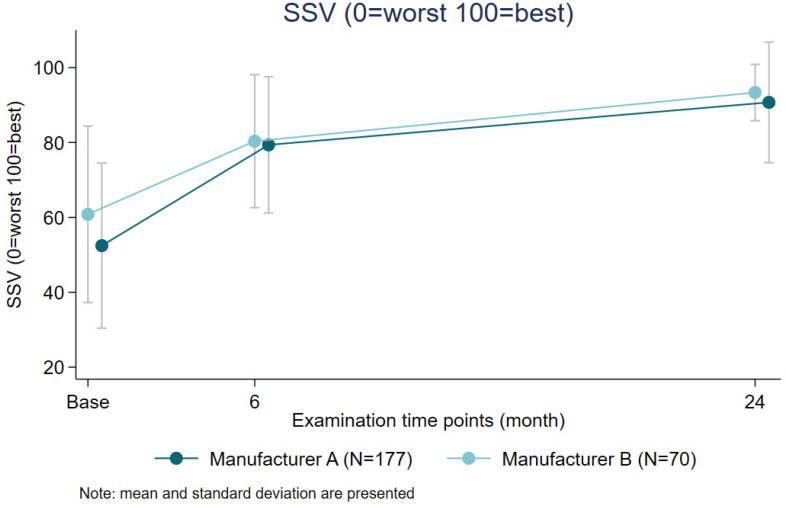
Comparison of SSV scores by anchor manufacturer over time.

There were no significant differences noted in the Subjective Shoulder Value (SSV) or quality of life measures between the two manufacturers (7.02, *p* = 0.11) (see Figure 2 and Figure 3).

In evaluating the clinical impact of different anchor configurations on the Oxford Shoulder Score (OSS), configurations 1 and 2 did not show statistically significant changes, with OSS decreases of 3.62 points (*p* = 0.13) and increases of 1.14 points (*p* = 0.73), respectively. However, configuration 3 demonstrated a small but statistically significant decrease of 4.15 points in OSS (*p* = 0.03).

**Figure 4:**
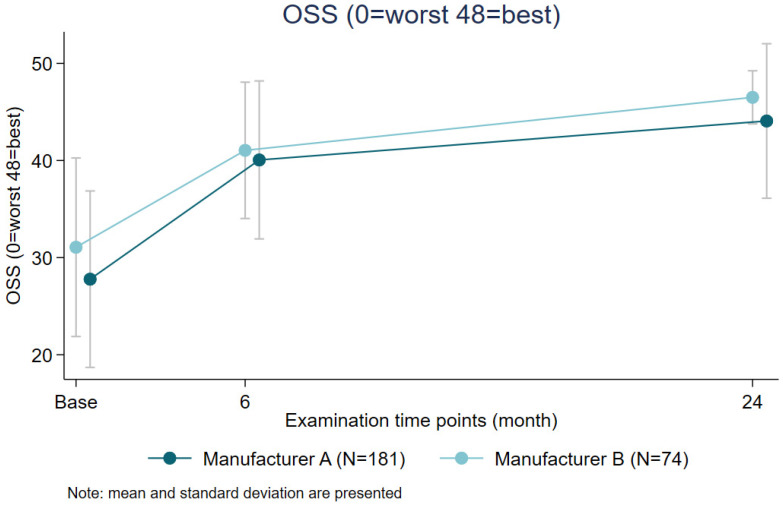
Comparison of OSS by anchor manufacturer over time.

A comparison between manufacturers revealed that using Manufacturer B anchors resulted in a statistically non-significant increase of 0.33 points in OSS (see Figure 4) compared to Manufacturer A anchors (*p* = 0.85).

Results from our regression analysis indicated that using anchors by Manufacturer A results in an incremental cost of CHF 93.68 per Adjusted Life Year gained compared to using Manufacturer B (reference category). The ICER calculation highlights a significant cost difference between the two manufacturer anchor types, with Manufacturer A being more costly per unit of effectiveness gained. The comparison between anchor manufacturers revealed that Manufacturer B was associated with significantly lower costs than anchors by Manufacturer A, with a mean overall cost difference of CHF 393.75 (*p* < 0.001).

Significant cost differences were also observed across anchor configurations when compared to the reference group (Configuration 4): Configuration 1 resulted in significantly reduced costs compared to the reference group, showing a cost decrease of CHF 253.72 (*p* < 0.001). Similarly, using configuration 2 decreased costs by CHF 254.51 (*p* < 0.05), and configuration 3 also showed a cost reduction of CHF 129.03 (*p* < 0.01).

## 7. Discussion

The findings comparing anchor configuration suggest that config. 3 could be associated with significantly poorer shoulder function as measured by the OSS, while the choice of manufacturer does not influence clinical outcomes. However, the minimum clinically important difference (MCID) for the OSS (6 points) was not reached, which indicated that the findings do not have a relevant clinical impact [12].

Despite this cost variance, no significant differences were noted in Incremental Cost-Effectiveness Ratios (ICERs), Quality-Adjusted Life Years, or quality of life measures. Similarly, the OSS and SSV scores revealed no notable disparities concerning the comparisons of anchor configurations from the manufacturer.

When analyzing cost-effectiveness by anchor configuration, configurations using fewer total anchors understandably demonstrated consistent superior cost-effectiveness profiles compared to the reference configuration 4. Configuration 1 showed the most favorable cost-effectiveness ratio, followed by configurations 2 and 3. These findings are in line with the previously published data from studies about the arthroscopic outpatient rotator cuff repair in North America, with a much higher impact reaching up to USD 2245 per anchor [13,14,15,16]. This seems to be the decisive factor, as patient-related factors were not shown to correlate well with operative costs [14,15].

Previous to TOE or double row (DR) reconstructions, single row (SR) was the standard configuration for rotator cuff repair. A study from 2012 highlighted the importance of how many re-tears of reconstructed rotator cuff need to be prevented in order for the higher costs, due to the additional anchors of the lateral row, to be justified [17]. While our study did not focus on structural outcomes and re-tear rates, the previously stated question on how to justify the use of additional anchors without evidence of added clinical value or Quality-Adjusted Life Years remains relevant to this day.

A recent study demonstrated a superior cost-effectiveness for knotless anchor configurations in ARCR, and the authors calculated a cost reduction of USD 492 due to a reduction in surgical time of approximately 41 min [7]. However, the authors also noted the higher overall implant costs in the knotless group due to additional anchors needed. Surgical time and implant costs remain the two most important factors in primary surgeries when trying to optimize cost-effectiveness in orthopedic practice. The results of our study support the integration of cost-effectiveness considerations into surgical decision-making for rotator cuff repair. A recent study has demonstrated the willingness of shoulder surgeons to practice cost-conscious healthcare, especially when there is an incentivization with a salary based on productivity and shared profits [18]. With rising healthcare-related costs in the leading European markets, healthcare systems and surgeons should consider these economic factors alongside clinical considerations when selecting anchor systems and configurations. Surgeons should evaluate whether more conservative anchor configurations can achieve adequate repair for specific tear patterns, and cost-effectiveness should also be integrated into surgical training curricula to promote value-based care.

From a value-based healthcare perspective, the equivalent clinical outcomes combined with significant cost differences provide clear guidance for optimizing resource utilization in rotator cuff surgery. These findings contribute to the growing body of evidence supporting evidence-based, cost-conscious surgical practice.

## 8. Limitations

Several limitations should be acknowledged: This retrospective analysis from a single institution may limit generalizability. The non-randomized nature of anchor selection introduces potential selection bias, although we controlled for key confounding variables. While controlling for surgeon variability, the design may limit external validity, as different surgeons may have varying preferences and proficiency with different anchor systems.

## 9. Conclusions

The findings of this study indicate that although clinical outcomes are generally consistent across various rotator cuff reconstruction scenarios, the cost implications differ significantly and are mainly attributed to the differing numbers of anchors required for each configuration and price setting by the manufacturer. The study underscores the importance of manufacturer and configuration selection in controlling healthcare costs in routine surgical procedures while maintaining quality patient care.

## Figures and Tables

**Table 1:** Patient characteristics by manufacturer (SD = standard deviation).

	Manufacturer A	Manufacturer B	
	*n* = 223	*n* = 94	*p*-Value
**Mean age at surgery (SD)**	60.1 (10.2)	60.2 (12.2)	0.976
**Sex (*n*, %)**			0.030
Female	103 (46)	31 (33)	
Male	120 (54)	63 (67)	
**Tear Severity (*n*, %)**			0.203
Partial tear	52 (23)	20 (21)	
Single full tear	75 (34)	43 (46)	
Two or three tendons (only one full)	51 (23)	18 (19)	
Massive tear	45 (20)	13 (14)	

## Data Availability

The datasets used and/or analyzed in the context of this study are available from the corresponding author on reasonable request.
